# A New Approach for Deep Gray Matter Analysis Using Partial-Volume Estimation

**DOI:** 10.1371/journal.pone.0148631

**Published:** 2016-02-04

**Authors:** Guillaume Bonnier, Tobias Kober, Myriam Schluep, Renaud Du Pasquier, Gunnar Krueger, Reto Meuli, Cristina Granziera, Alexis Roche

**Affiliations:** 1 Advanced Clinical Imaging Technology, Siemens Healthcare IM BM PI, Lausanne, Switzerland; 2 LTS5, École Polytechnique Fédérale de Lausanne, Lausanne, Switzerland; 3 Department of Clinical Neurosciences, LREN and Neuroimmunology Laboratory, Centre Hospitalier Universitaire Vaudois (CHUV), Lausanne, Switzerland; 4 Department of Radiology, Centre Hospitalier Universitaire Vaudois (CHUV), Lausanne, Switzerland; 5 Siemens Medical Solutions USA IM MR COL NEZ, Burlington, MA, United States of America; University Health Network and University of Toronto, CANADA

## Abstract

**Introduction:**

The existence of partial volume effects in brain MR images makes it challenging to understand physio-pathological alterations underlying signal changes due to pathology across groups of healthy subjects and patients. In this study, we implement a new approach to disentangle gray and white matter alterations in the thalamus and the basal ganglia. The proposed method was applied to a cohort of early multiple sclerosis (MS) patients and healthy subjects to evaluate tissue-specific alterations related to diffuse inflammatory or neurodegenerative processes.

**Method:**

Forty-three relapsing-remitting MS patients and nineteen healthy controls underwent 3T MRI including: (i) fluid-attenuated inversion recovery, double inversion recovery, magnetization-prepared gradient echo for lesion count, and (ii) T1 relaxometry. We applied a partial volume estimation algorithm to T1 relaxometry maps to gray and white matter local concentrations as well as T1 values characteristic of gray and white matter in the thalamus and the basal ganglia. Statistical tests were performed to compare groups in terms of both global T1 values, tissue characteristic T1 values, and tissue concentrations.

**Results:**

Significant increases in global T1 values were observed in the thalamus (*p = 0*.*038*) and the putamen (*p = 0*.*026*) in RRMS patients compared to HC. In the Thalamus, the T1 increase was associated with a significant increase in gray matter characteristic T1 (*p = 0*.*0016*) with no significant effect in white matter.

**Conclusion:**

The presented methodology provides additional information to standard MR signal averaging approaches that holds promise to identify the presence and nature of diffuse pathology in neuro-inflammatory and neurodegenerative diseases.

## Introduction

Magnetic resonance (MR) imaging provides *in vivo* information about brain tissue integrity [1]. The MR signal varies across tissue types because gray matter (GM) contains a prevalence of cell bodies (neurons, glial cells, etc....) and iron, while white matter (WM) is for the majority constituted by neuronal fibers (myelinated and unmyelinated axons) [2,3]. Nevertheless, as cell bodies are common in WM, a certain amount of neuronal fibers is also present in cortical and sub-cortical GM [4]. The variation of the MR signal due to the mixture of different tissues in the same region is known as the “partial volume” (PV) effect. In deep gray matter nuclei (DGMN), the PV effect modulates the MR signal across and throughout the anatomical structures due to presence of neuronal fibers in varying amount. The globus pallidus shows higher intensity in T1-weighted images (close to WM T1) than other DGMN, due to its relatively higher myelin content [5]. On the other hand, the putamen is characterized by higher T1 intensity in its caudal part when compared to the rest of its structure, due to the presence of myelinated axons [6].

Because of this, the analysis of the GM and WM alterations in the DGMN is challenging. Standard approaches based on global signal averaging compare mean intensities over regions of interest. While such methods may detect changes across different populations, they do not provide enough information to attribute such changes to specific changes in the GM or WM components of the MR signal.

In the recent past, a number of MRI approaches based on the combination of different contrasts, have been proposed to visualise cortical myelination and differentiate the cellular from neuronal fibers component in the brain cortex. *Glasser et al*. computed the ratio between T1- and T2-weighted MR images as a measure of cortical myelin content [7], while *Grydeland* combined this ratio with diffusion MRI information to achieve more accurate results [8]. More recently, *Shafee et al*. performed cortical myelin content estimation using a mixture model of GM and WM in both T1- and T2-weighted images. As to deep GM structures (thalamus and basal ganglia), there have been a few attempts to separate their tissue components based on advanced MRI techniques like relaxometry and magnetization transfer imaging [6] or quantitative susceptibility mapping [9]. These approaches provide measures directly linked with the biological properties of the tissue. The longitudinal relaxation time T1 which is very sensitive to the structural coherence of the tissue, provides a very good contrast between the GM and WM. The magnetization transfer imaging is based on the signal of the macromolecules composing the tissue, and is particularly sensitive to myelin [10]. These multi-contrast approaches have the potential to provide evidence of demyelination processes, but may be insensitive to changes in GM properties, which may occur in others pathologies.

In this work, we aim to characterize both the GM and WM components of the DGMN by disentangling their respective contributions to the MR signal. We use a method of partial volume estimation previously described and validated in (Roche and Forbes, MICCAI 2014) [11] by comparison with the MNI BrainWeb phantom (http://brainweb.bic.mni.mcgill.ca/brainweb/).

In the sequel, we describe our tissue separation method, and further present its application to a group comparison study between early relapsing remitting multiple sclerosis (RRMS) patients and healthy controls.

## Method

### Population

We studied nineteen healthy controls (HC, 33 ± 9.3 years mean ± standard deviation (SD), 11 women and 8 men, gender ratio: 1.7) and forty-three RRMS patients (35.2 ± 10 years mean ± SD, 27 women and 16 men, gender ratio: 1.4).

Subjects gender ratio and age showed no significant differences between controls and RRMS patients group.

All patients were scanned less than 6 years after the initial symptoms (33 ± 20 months, range 2–70 months) and the disease diagnosis (27 ± 17 months, range 0–59 months). Patients had a low lesion load considering the early stage of the disease (mean total lesion volume per patient: 5 ± 2 mL).

Thirty patients were under immunomodulatory treatment (high dosage interferon beta or fingolimod) for at least 3 months, the rest of the patients were not under treatement at the time of the MRI. No patient had received corticosteroid therapy within the 3 months preceding the enrollment.

The ethics committee of the University Hospital of Lausanne (CHUV) approved the study. Written informed consent was obtained from each subject.

### Multiple sclerosis data acquisition

All subjects underwent the following protocol: (i) 3D magnetization-prepared acquisition with gradient echo (MPRAGE) (TR/TE = 2300/2.89 ms, voxel size = 1.0x1.0x1.2 mm^3^, FoV = 256x256x256, acquisition time = 5:12 min), (ii) a prototype 3D magnetization-prepared 2 acquisitions with gradient echo (MP2RAGE) [12] (TR/TE = 5000/2.89 ms, voxel size = 1x1x1.2 mm^3^, FoV = 256x256x256 acquisition time = 8:22 min), (iii) a high-resolution 3D fluid-attenuated inversion recovery (FLAIR) (TR/TE/TI = 5000/394/1800 ms, voxel size = 1x1x1.2 mm^3^, FoV = 256x256x256, acquisition time = 6:27 min); and (iv) a 3D double inversion recovery (DIR) (TR/TE/TI = 10000/218/3650 ms, voxel size = 1x1x1.2 mmm^3^, FoV = 256x256×256, acquisition time = 12:52 min). All acquisitions were performed on a 3T MAGNETOM Trio (Siemens, Erlangen, Germany) equipped with a 32-channel head coil. The MPRAGE sequence was used for atlas-based segmentation of the DGMN. The MP2RAGE, FLAIR and DIR sequences were used to detect and segment MS lesions in the brain. The MP2RAGE sequence also provided a whole-brain T1 relaxometry map [12].

A group of 9 healthy subjects (30.2 ± 5 years mean ± SD, 6 women and 3 men) also elected to participate in the 7T study, underwent a MP2RAGE sequence at 7T. A visual inspection of image quality was performed for all subjects.

### Image processing

#### Regions-of-interest extraction

We applied the MorphoBox software [13] to the MPRAGE volumes to segment the following deep GM regions: thalamus, caudate, putamen and pallidum. MorphoBox automatically delineates ROIs by performing a non-rigid registration to a template, previously manually defined by a neurologist and two neuroradiologists by consent. In order to work only with normal-appearing tissue, we excluded MS lesions from each DGMN. The lesions were manually identified and delineated by an experienced neurologist (CG) and a radiologist (DR) using FLAIR, DIR and MP2RAGE images. Then lesions masks were created using the methodology previously reported in [14].

#### Concentration map and tissue intensities

The intensity of a voxel in an MR image depends on the specific imaging technique used and the type of molecules present in the voxel volume. In a T1 relaxometry map, the voxel intensity represents the longitudinal relaxation time of the tissue and is driven by the density and type of molecules that compose the tissue. The microstructure of the DGMN is mainly characterized by neurons and glial cells (GM component) as well as neuronal fibers (WM component) [2], which have a different impact on the MRI signal. To separate GM and WM components in a DGMN and estimate their respective contributions to the T1 intensity, we applied a partial volume estimation algorithm developed by *Roche A*. *et al*. [11], which operates from a model that describes each voxel intensity as the sum of GM and WM characteristic intensities (which are global to DGMN) weighted by their respective concentrations (which are local), with Gaussian noise:

$$y_{i}=\mu_{GM}C_{GM}+\mu_{WM}C_{WM}+\varepsilon_{i}$$

*with*

$$\varepsilon_{i}=N\left(0,\;\sigma \right)$$

where *y*_*i*_ is the intensity of a voxel *i*, *C*_*GM*_ and *C*_*WM*_ are the associated concentrations of GM and WM, *μ*_*GM*_ and *μ*_*WM*_ the characteristic tissue intensities, i.e. the intensities corresponding to 100% of GM and WM, respectively; *ε*_*i*_ represents the noise and *σ* its standard deviation, which is also estimated by the PV algorithm.

After we initialized both *μ*_*GM*_ and *μ*_*WM*_ to standard T1 values at 3T for GM (1350 ms) and WM (850 ms) [12], the algorithm iteratively estimated GM and WM concentrations (*C*_*GM*_, *C*_*WM*_) and their characteristic intensities *μ*_*GM*_ and *μ*_*WM*_ for each voxel: (Step 1) the algorithm estimates tissue concentration maps assuming tissue characteristic values *μ*_*GM*_ and *μ*_*WM*_; (Step 2) it re-computes the characteristic intensities *μ*_*GM*_ and *μ*_*WM*_ of GM and WM based on the concentration maps previously estimated. These two steps were iterated 10 times to provide stable estimates of both GM and WM concentration maps and T1 characteristic intensities for each DGMN. See an example of GM concentration map in the supplementary data (S1 Fig).

### Qualitative assessment

In order to illustrate the extracted GM and WM components, we compared GM concentration maps estimated for 3 healthy subjects at 3T and 7T with histological slices obtained from one healthy subject at the level of the DGMN [15,16]. The 3 healthy subjects (32.2 ± 3 years mean ± SD, 2 women and 1 man) were chosen randomly among the 9 HC who were scanned at 3T and 7T.

**Fig 1:**
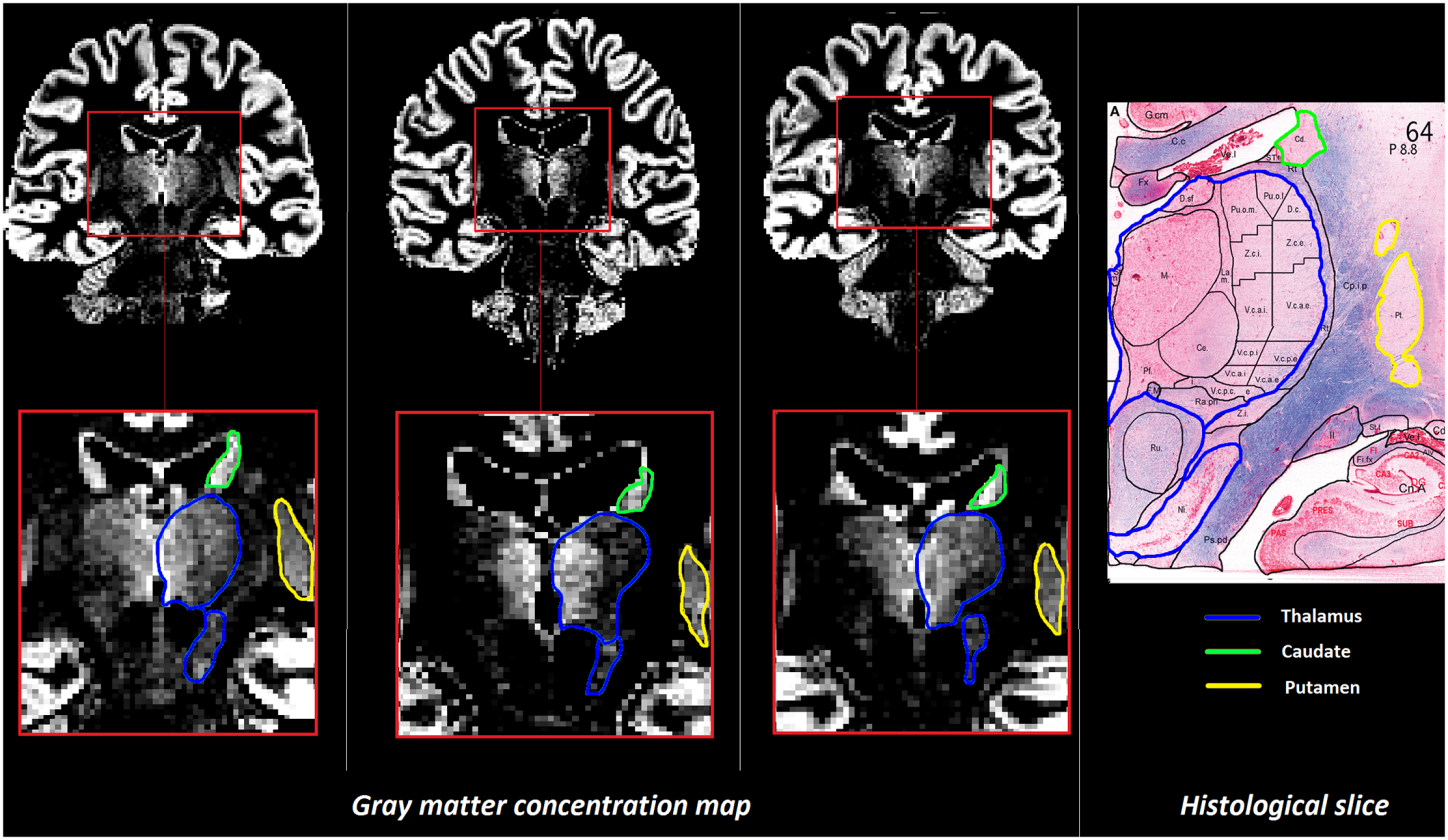
Comparison between GM concentration map of 3 healthy subjects at 3T and an histological slice [15]. Histological data show outline of nuclei of the human thalamus on a coronal Myelin-Nissl-stained section (*right*). The histologic sections are taken at the level of the sensory thalamus. Thalamic nuclei are outlined using the terminology in Schaltenbrand’s atlas according to Hassler. The concentration maps were estimated using the whole-brain T1 maps of 3 healthy subjects randomly chosen. In the zoomed area and the histological slice, the DGMN are delineated as follows: thalamus in blue, putamen in yellow, caudate in green.

The histological images were stained using Nissl to highlight the cell bodies, and Luxol blue to show myelin of axons (Fig 1) [15].

### Group comparison analysis

We subsequently performed a group analysis to compare RRMS patients and controls using: (i) a multivariate analysis of the variances (MANOVA) of the estimated pooled T1 intensities of GM and WM as well as the ratio between GM and WM concentrations (*C*_*GM*_ / *C*_*WM*_), (ii) a global averaging technique of the T1 intensity.

All statistical analysises were corrected for multiple comparison using Bonferroni correction or the family-wise error rate [17], and age and sex were used as covariates.

The MANOVA was performed using R software (http://www.R-project.org) and tested the following H0 hypothesis: there are no differences between RRMS patients and controls in the WM and GM pooled intensities (*μ*_*GM*_ and *μ*_*WM*_) and in the concentration ratio (*C*_*GM*_ / *C*_*WM*_) of thalamus, caudate, putamen, and pallidum. In order to identify the contribution of GM and WM characteristic intensities and concentration to the differences observed with the multivariate analysis, we performed two-tailed permutation-based two-sample t-tests between RRMS patients and controls on each component (*μ*_*GM*_, *μ*_*WM*_, *C*_*GM*_ / *C*_*WM*_). Age and gender were used as covariates.

The global averaging approach consisted in analysing mean T1 relaxation times in each DGMN by performing a two-sample t-tests between RRMS patients and controls.

## Results

### Qualitative assessment

Qualitative comparison between the concentration maps obtained from 3T data with two histological slices obtained from *Sadikot* and *Chakravarty* [15,16] showed a number of similar details. The first slice shows the histology of the thalamus, the caudate and the putamen (Fig 1): neuronal nuclei in red, and myelin in blue. While the concentration of nuclei in the putamen and caudate appears homogeneous, the thalamus shows a variable amount of nuclei which gradually increases towards the ventricles. The concentration maps estimated from the T1 map at 3T show very similar homogeneity in the GM concentration of the caudate (delineated in green) and putamen (delineated in yellow), while the thalamus (delineated in blue) exhibits an increase of GM concentration going from the border of the structure to its center. The red coloration in the histological slice shows a higher concentration of neuronal nuclei in the caudate than in the putamen. The GM concentration map reflects this difference as the caudate appears brighter than the putamen for the 3 subjects. We also noticed a higher GM concentration in caudate than in the lateral part of the thalamus in the GM concentration maps, while coloration appears less red in histological data of the lateral part of the thalamus which may reflect presence of myelin. Furthermore, the concentration maps visualized the red nucleus and the locus niger as reported by histological data.

**Fig 2:**
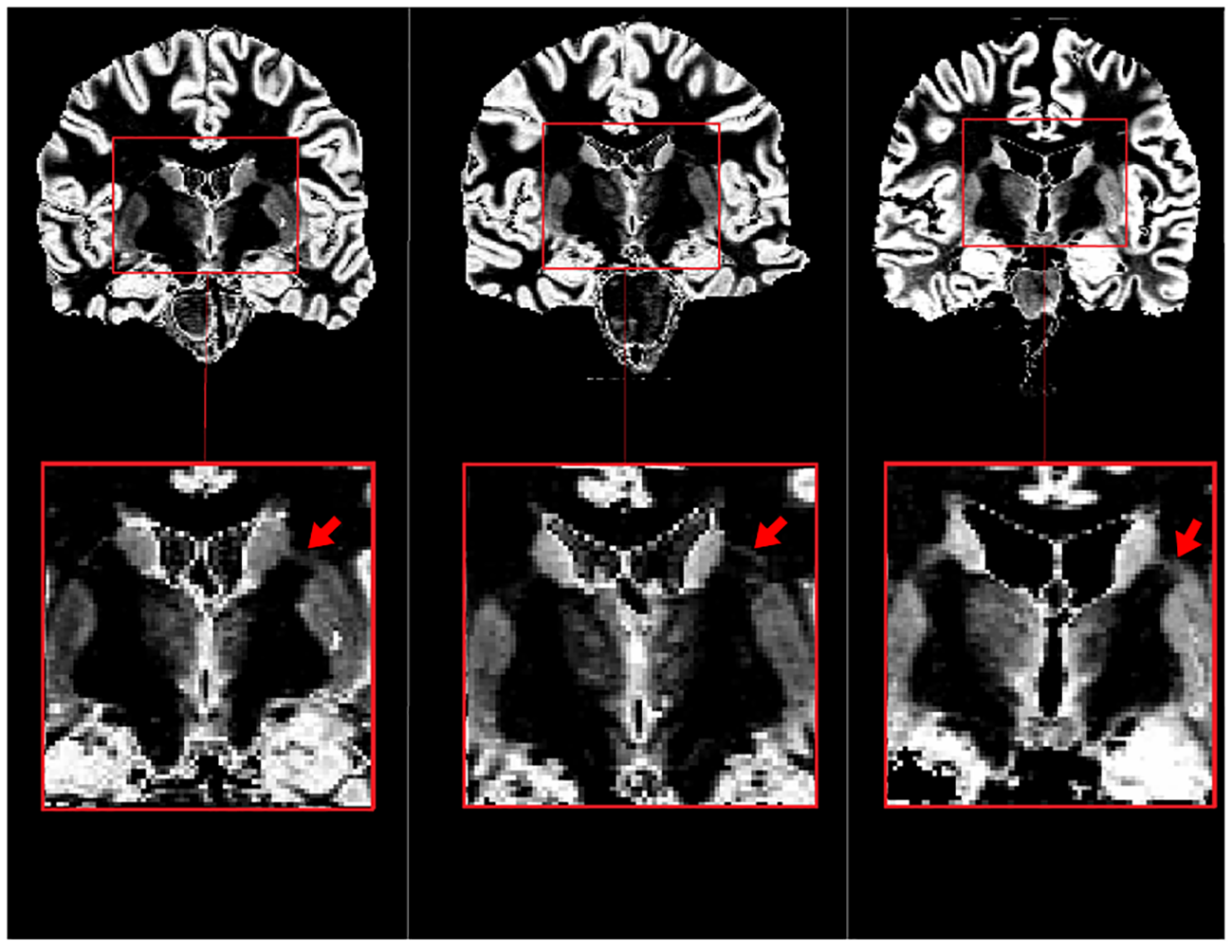
Comparison between GM concentration map of 3 healthy subjects at 7T and an histological slice from a healthy human subject [16]. The concentration map were estimated using the whole-brain T1 map. In the zoomed area of GM concentration map we can observe the gray matter bridge present in the histological slice between the caudate and the putamen.

Similar observations are made in others slices from the estimated concentration maps of 3 HC scanned at 7T compared with histological staining [16]. Due to the higher spatial resolution at 7T (Fig 2), images revealed an additional detail, a GM bridge between the caudate and the putamen, which is clearly observable on stained images proposed in [16].

### Group comparison using MANOVA and global averaging

The multivariate analysis of T1 characteristic intensities and concentrations ratio revealed a significant difference in the thalamus of RRMS patients (*p*_*Corrected*_ *= 0*.*0016*).

**Fig 3:**
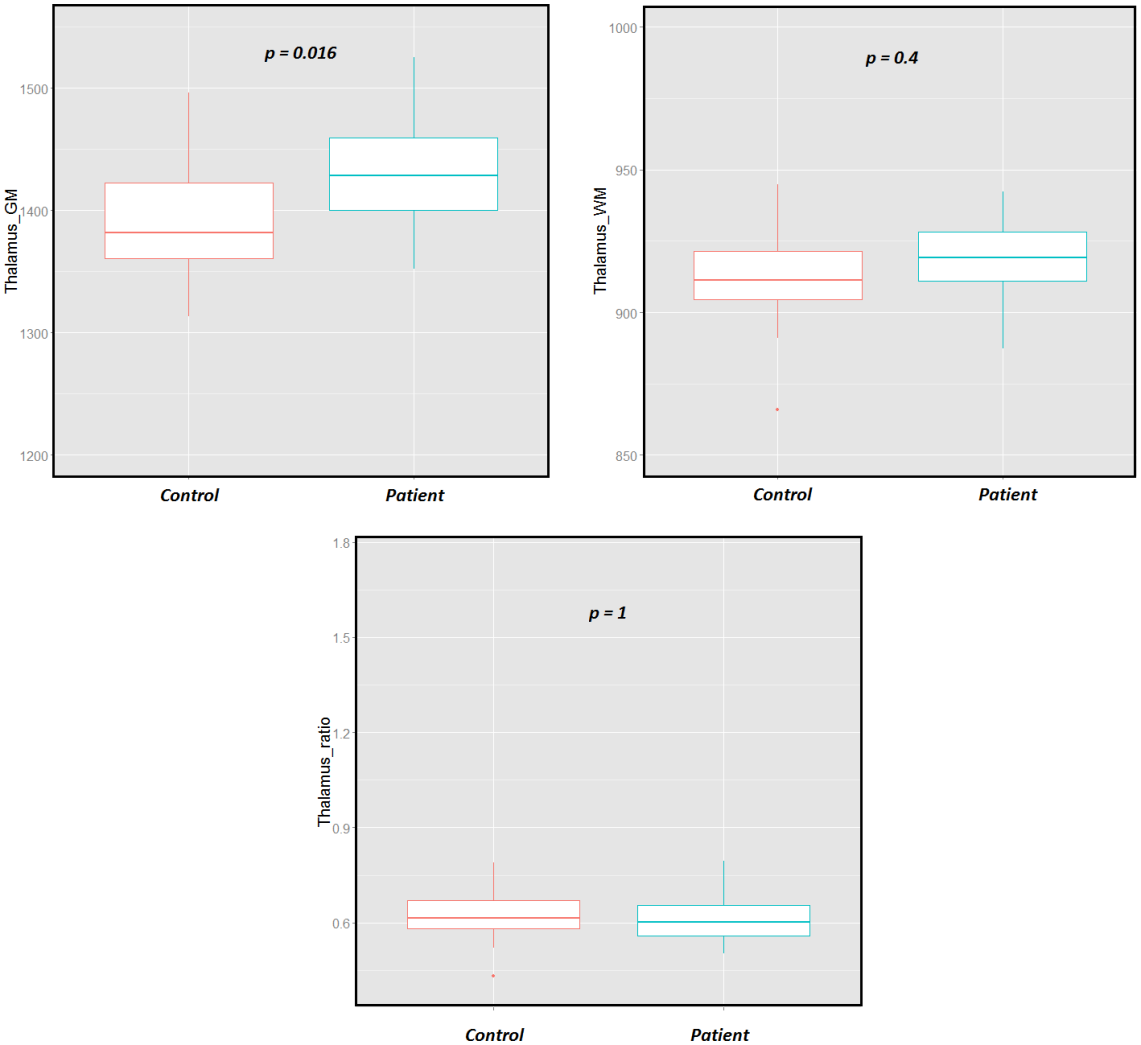
Boxplots of the WM and GM of the thalamus for RRMS patients and controls using the proposed PV method. It shows significant increase in the T1 of the GM components between the RRMS patients and HC.

The analysis of the tissue T1 pooled intensities and concentrations showed a significant increase of 3% of the GM pooled intensity (*μ*_*GM*_) in the RRMS patients (*Controls*: *μ*_*GM*_ *= 1389 ± 47 ms; Patients*: *μ*_*GM*_ *= 1427 ± 40 ms; p = 0*.*016*), while the WM pooled intensity (*μ*_*WM*_) (0.6%) and the concentration ratio (-1%) were not found significantly different (*Controls*: *μ*_*WM*_ *= 912 ± 18 ms*, *μ*_*ratio*_ *= 0*.*62 ± 0*.*08; Patients*: *μ*_*WM*_ *= 918 ± 14 ms*, *μ*_*ratio*_ *= 0*.*61 ± 0*.*07*) (Fig 3).

**Fig 4:**
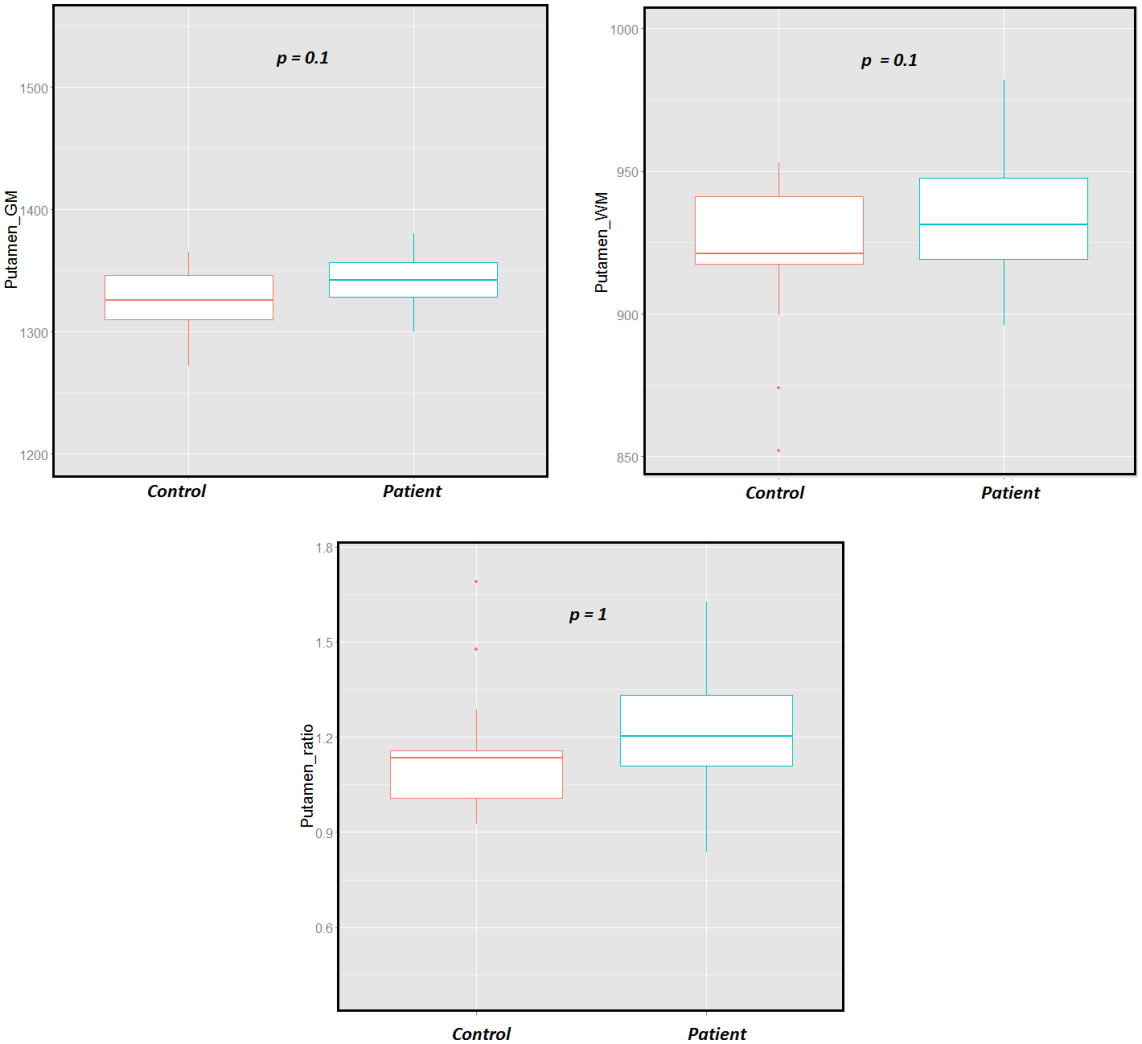
Boxplots of the WM and GM of the putamen for RRMS patients and controls using partial volume method. This method showed no significant increase in the T1 of the GM and WM components neither concentration ratio between the RRMS patients and HC.

The putamen, caudate and pallidum did not show any significant changes between RRMS patients and HC using the multivariate analysis (Fig 4).

**Fig 5:**
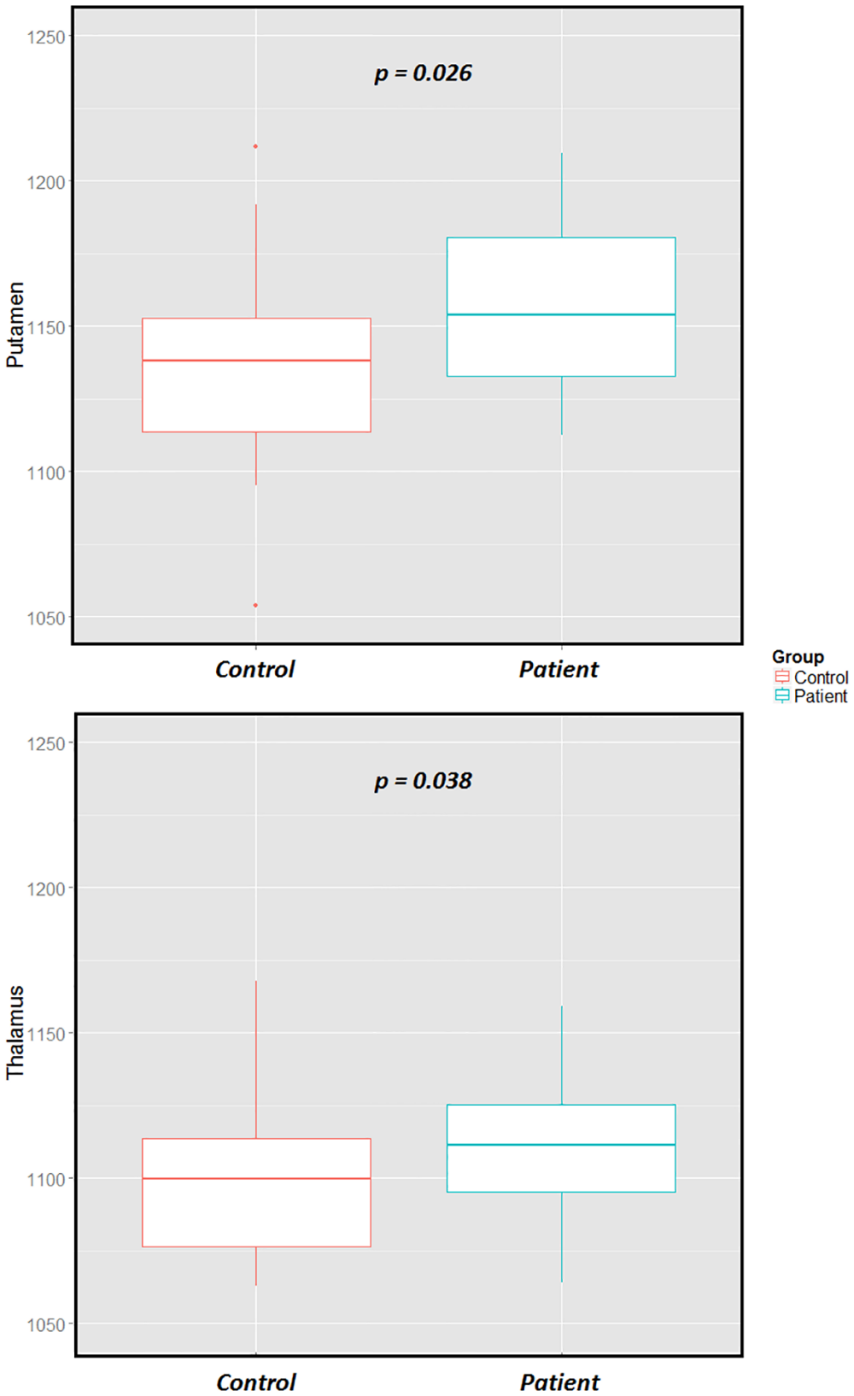
Boxplot of mean T1 in the thalamus and putamen using global averaging method. This method showed significant increase of T1 in both ROIs by RRMS patients compared with HC.

The global averaging analysis showed significant differences in the thalamus but also in the putamen between RRMS patients and HC. In both structures, we observed a significant T1 increase in RRMS patients compared with HC (thalamus: *p = 0*.*038; HC*: *μ*_*T1*_ *= 1094 ± 33 ms; RRMS*: *μ*_*T1*_ *= 1110 ± 22 ms; putamen*: *p = 0*.*026; HC*: *μ*_*T1*_ *= 1136 ± 35 ms; RRMS*: *μ*_*T1*_ *= 1156 ± 27 ms*, Fig 5*)*.

## Discussion

This study presents a new approach to disentangle GM and WM components in regions affected by partial volume in MRI like the thalamus and the basal ganglia.

The proposed methodology provides quantitative estimations of GM and WM concentrations and T1 relaxometry times by pooling intensity values that are characteristic for GM and WM within a particular region. This opens new perspectives for the evaluation of the nature of pathological processes.

In this study, we used an in-house algorithm [11] to estimate tissue concentrations and characteristic intensities. The qualitative comparison of the estimated tissue concentration maps with histological data showed remarkable similarities, including the heterogeneity of cellular concentrations in the thalamus, and the variable amount of myelin among the DGMN. In summary, these findings provide qualitative evidence of the relevance of the partial volume estimation proposed by *Roche et al*. [11] in deep GM regions.

Group analysis using MANOVA based on T1 relaxation times and concentrations of each deep GM nucleus showed significant differences in the thalamus between patients and controls (*p = 0*.*0016*). The analysis of the thalamus further revealed that the observed differences were due to an increase of the intrinsic T1 value of the GM in RRMS patients (*p = 0*.*016; 3%*), with no significant changes in the T1 of the WM component nor the WM/GM concentration ratio. The global averaging method revealed significant changes in the thalamus with a less significant p-value *(p = 0*.*038*) than the MANOVA, but it also detected significant changes in the putamen (*p = 0*.*026*), while the MANOVA showed no significance (*p = 0*.*4*). In addition, the analysis of tissue T1 pooled intensities and concentrations in the putamen did not show significant changes between RRMS patients and controls (*p*_*GM_T1*_ *= 0*.*1; p*_*WM_T1*_ *= 0*.*16; p*_*ratio*_ *= 0*.*13)*. This result shows that an accumulation of non-significant small effects in each component (GM and WM pooled intensities and concentrations increased in the putamen) can become significant when combined. This observation suggests that although it does not identify the source of intensity changes, the global averaging method appears more sensitive to detect changes than the MANOVA. Nevertheless, considering the increased number of features (3 features instead of 1) analyzed in the MANOVA a larger cohort would be required to assign observed differences in average T1 values to specific changes in GM or WM.

As all patients were under the same medications at scan time and the use of covariates such as age, gender in our analyses, we stated that the differences observed between patients and HC were mainly due to pathological effects. Clinically these results suggest that thalamic pathology in our population of early RRMS patients mainly affects the GM pool, while in the putamen the WM and GM components appeared equally affected. From a pathophysiological viewpoint, these findings might reflect the substantial neuronal damage that has been described in the thalamus of multiple sclerosis patients in previous neuropathological studies [18–22]. *Cifelli et al*. indeed observed thalamic volume reduction combined with *N*-acetylaspartate concentration and neuronal density decrease in thalamus of advanced MS patients [18], while a histo-pathological study showed evidence of deep GM structures atrophy correlated with neuronal loss by analyzing hippocampal autopsy tissue of MS patients [20]. Neuronal injury in the thalamus may be the consequence of wallerian or transynaptic degeneration and of injury from cytokines, oxidative stress, glutamate excitotoxicity, and CD8 T-cell–citotoxicity [19,22]. Although the causes of GM injuries in MS remains poorly understood, the GM pathologies in DGMN appear throughout all disease stages and involves at a time inflammatory and neurodegenerative processes [21]. Interestingly, the fact that we did not detect changes in the GM concentration might indicate a stage of damage that precedes a loss of GM in the end-thalamic volume [23], which may provide in the f**u**ture more info**r**mation on the pathogenesis of GM alteration in DGMN.

Although global averaging methods remain sensitive ways to perform region-wise group comparisons, the combination of these methods with the proposed PV estimation approach provided additional information about the processes underlying thalamic alterations in our cohort of MS patients. The presented methodology renders possible an in-depth characterization of DGMN tissue that helps to identify diffuse inflammatory or neurodegenerative pathologies affecting the basal ganglia and the thalamus.

## Supporting Information

S1 Fig Comparison between 2 axial slices of GM concentration maps (first row) and Statistical Parametric Mapping (SPM) GM probabilistic maps (second row) at 3T. The SPM map appears more binary, and some parts (pointed out by red arrows) of the thalamus and putamen disappear compared with concentration maps.
(DOCX)
