# Supplementary material for: A New Approach for Deep Gray Matter Analysis Using Partial-Volume Estimation
Source: PLoS One. 2016 Feb 4;11(2):e0148631. doi: 10.1371/journal.pone.0148631 (PMC4741419; doi:10.1371/journal.pone.0148631)
Supplement: S1 Fig — The SPM map appears more binary, and some parts (pointed out by red arrows) of the thalamus and putamen disappear compared with concentration maps. (DOCX) [file pone.0148631.s001.docx]

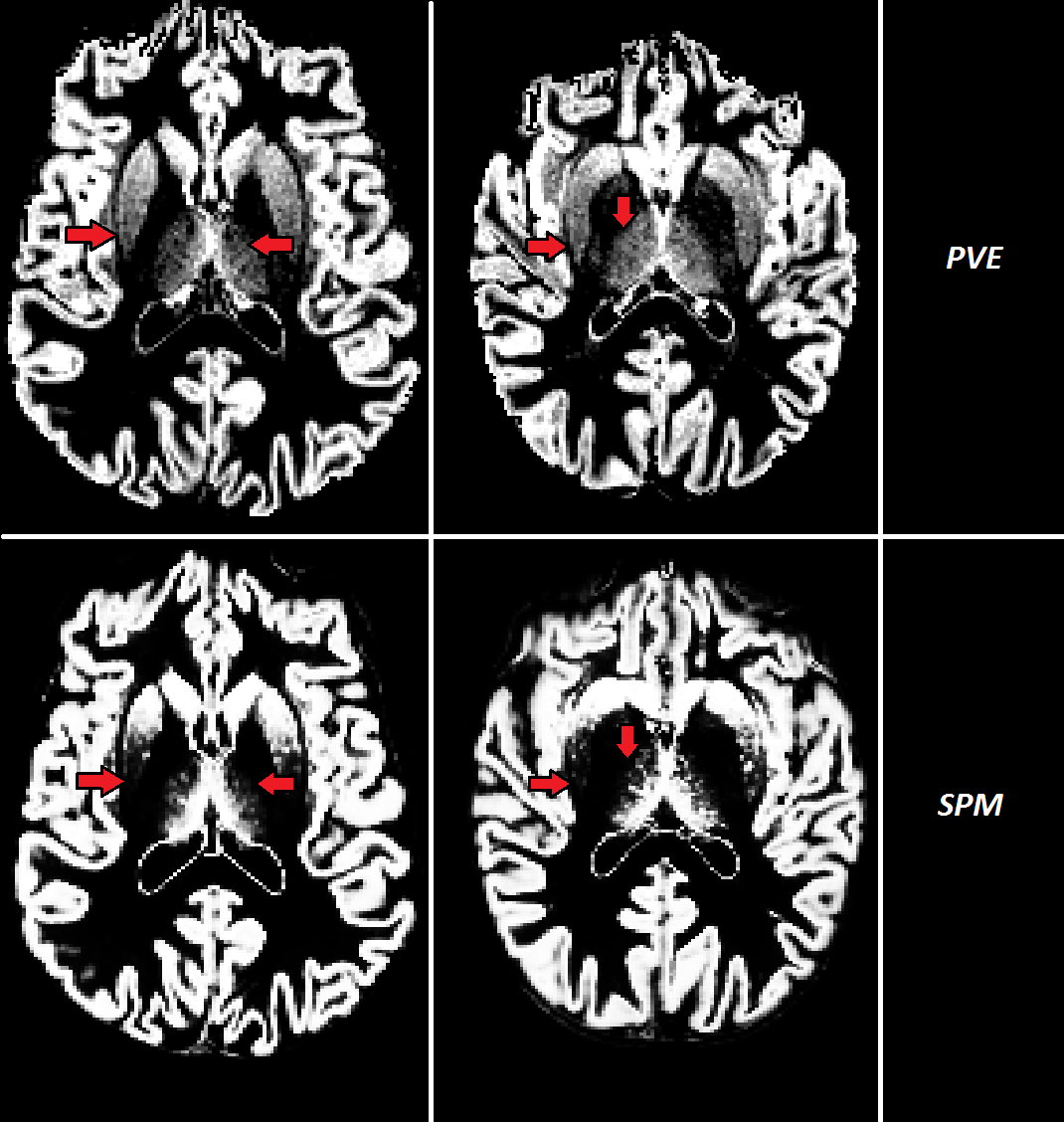


Comparison between 2 axial slices of GM concentration maps (first row) and Statistical Parametric Mapping (SPM) GM probabilistic maps (second row) at 3T. The SPM map appears more binary, and some parts (pointed out by red arrows) of the thalamus and putamen disappear compared with concentration maps.
